# Supplementary material for: The Accumulation of Progerin Underlies the Loss of Aortic Smooth Muscle Cells in Hutchinson-Gilford Progeria Syndrome
Source: bioRxiv. 2024 Nov 3:2024.10.29.620896. Preprint. [Version 1] doi: 10.1101/2024.10.29.620896 (PMC11565845; doi:10.1101/2024.10.29.620896)
Supplement: Supplement 1 [file NIHPP2024.10.29.620896v1-supplement-1.pdf]

bioRxiv preprint doi: https://doi.org/10.1101/2024.10.29.620896; this version posted November 3, 2024. The copyright holder for this preprint (which was not certified by peer review) is the author/funder, who has granted bioRxiv a license to display the preprint in perpetuity. It is made available under aCC-BY 4.0 International license.

Supplementary Materials

The Accumulation of Progerin Underlies the Loss of Aortic Smooth Muscle Cells in Hutchinson-Gilford Progeria Syndrome

Paul H. Kim, Joonyoung R. Kim, Patrick J. Heizer, Hyesoo Jung, Yiping Tu, Ashley Presnell, Julia Scheithauer, Rachel G. Yu, Stephen G. Young, and Loren G. Fong

Contents

Materials and Methods

Figures S1–S7.

Tables S1–S3.

bioRxiv preprint doi: https://doi.org/10.1101/2024.10.29.620896; this version posted November 3, 2024. The copyright holder for this preprint (which was not certified by peer review) is the author/funder, who has granted bioRxiv a license to display the preprint in perpetuity. It is made available under aCC-BY 4.0 International license.

Materials and Methods

Culture of aortic smooth muscle cells. Immortalized mouse aortic smooth muscle cells (SMCs) were purchased from ATCC (#CRL-2797) and cultured in DMEM (Invitrogen) supplemented with 10% (v/v) fetal bovine serum (HyClone), 1× nonessential amino acids, 2 mM glutamine, 1 mM sodium pyruvate, and 0.2 mg/ml G418 at 37°C with 5% CO_2.

Western blotting. Urea-soluble protein extracts from tissues and SMCs were prepared as described previously (*17, 21*). The extracts were mixed with LDS sample buffer (Invitrogen) and heated at 70°C for 10 min. The samples were size-fractionated on 4–12% gradient polyacrylamide Bis-Tris gels (Invitrogen) and transferred to nitrocellulose membranes. The membranes were blocked with Odyssey Blocking solution (LI-COR Bioscience, Lincoln, NE) for 1 hour at RT and incubated with primary antibodies at 4°C overnight. After washing the membranes with PBS containing 0.2% Tween-20 (3 times for 10 min each), they were incubated with infrared (IR) dye–labeled secondary antibodies at RT for 1 hour. Membranes were washed with 0.2% PBS-T (3 times for 10 min each). The IR signals were quantified with an Odyssey infrared scanner (LI-COR Biosciences). The antibodies and concentrations are listed in **Supplementary Table 1**.

Quantitative real time-PCR. Total RNA was extracted with the RNeasy kit (Qiagen) and treated with DNase I (Ambion) according to the manufacturer's recommendation. RNA was reverse-transcribed with random primers using SuperScript III cDNA Synthesis Kit (Invitrogen). cDNA samples were diluted in nuclease-free water and stored at –80°C. RT-PCR reactions were performed on a QuantStudio5 system (ThermoFisher Scientific) with SYBR Green PCR Master Mix (Bioland). Transcript levels were calculated by the comparative cycle threshold method and normalized to cyclophilin A expression. All primers used in the experiments are listed in **Supplementary Table 2**.

Doxycycline (Dox)-inducible expression in SMCs. SMCs harboring Dox-inducible pTRIPZ expression vectors for human prelamin A and human progerin have been described previously (*15, 21*). All plasmids were verified by DNA sequencing. Packaging of lentivirus and transduction of cells were performed by UCLA's Vector Core. Transduced cells were selected with 3 μg/ml

bioRxiv preprint doi: https://doi.org/10.1101/2024.10.29.620896; this version posted November 3, 2024. The copyright holder for this preprint (which was not certified by peer review) is the author/funder, who has granted bioRxiv a license to display the preprint in perpetuity. It is made available under aCC-BY 4.0 International license.

puromycin for two weeks; individual clones were isolated by limiting dilution in 96-well plates. Clones were screened by western blotting and immunofluorescence staining. A minimum of 2 clones were isolated for each cell line.

Constitutive expression of nuclear localized GFP and lamins in SMCs. SMCs expressing green fluorescent protein (GFP) with a nuclear localization signal (nls-GFP) have been described previously (*21, 31*). Constitutive expression of human versions of prelamin A and progerin in SMCs was performed by transducing SMCs with pCLNR (#17735; Addgene) retroviral expression plasmids. The prelamin A and progerin cDNAs were subcloned into *Hind*III and *Not*I sites of pCLNR by In-Fusion cloning (Takara Bio). Human prelamin A cDNA was amplified with forward primer 5′-GCTAGCGAATTATGGAGACCCCGTCCCAGC-3′ and reverse primer 5′-GATCCTTGCGGCCTTACATGATGCTGCAGT-3′; human progerin cDNA was amplified with forward primer 5′-GCTAGCGAATTATGGAGACCCCGTCCCAGC-3′ and reverse primer 5′-CAGATCCTTGCGGCCTTACATGATGCTGCA-3′. All plasmid DNAs were prepared with Maxiprep kit (Qiagen) and verified by DNA sequencing. Packaging of virus and viral transduction were performed by UCLA's Vector Core. Transduced cells were selected with 3 μg/ml blasticidin or puromycin for two weeks; individual clones were isolated by limiting dilution. A minimum of 2 clones were isolated for each cell line.

bioRxiv preprint doi: https://doi.org/10.1101/2024.10.29.620896; this version posted November 3, 2024. The copyright holder for this preprint (which was not certified by peer review) is the author/funder, who has granted bioRxiv a license to display the preprint in perpetuity. It is made available under aCC-BY 4.0 International license.

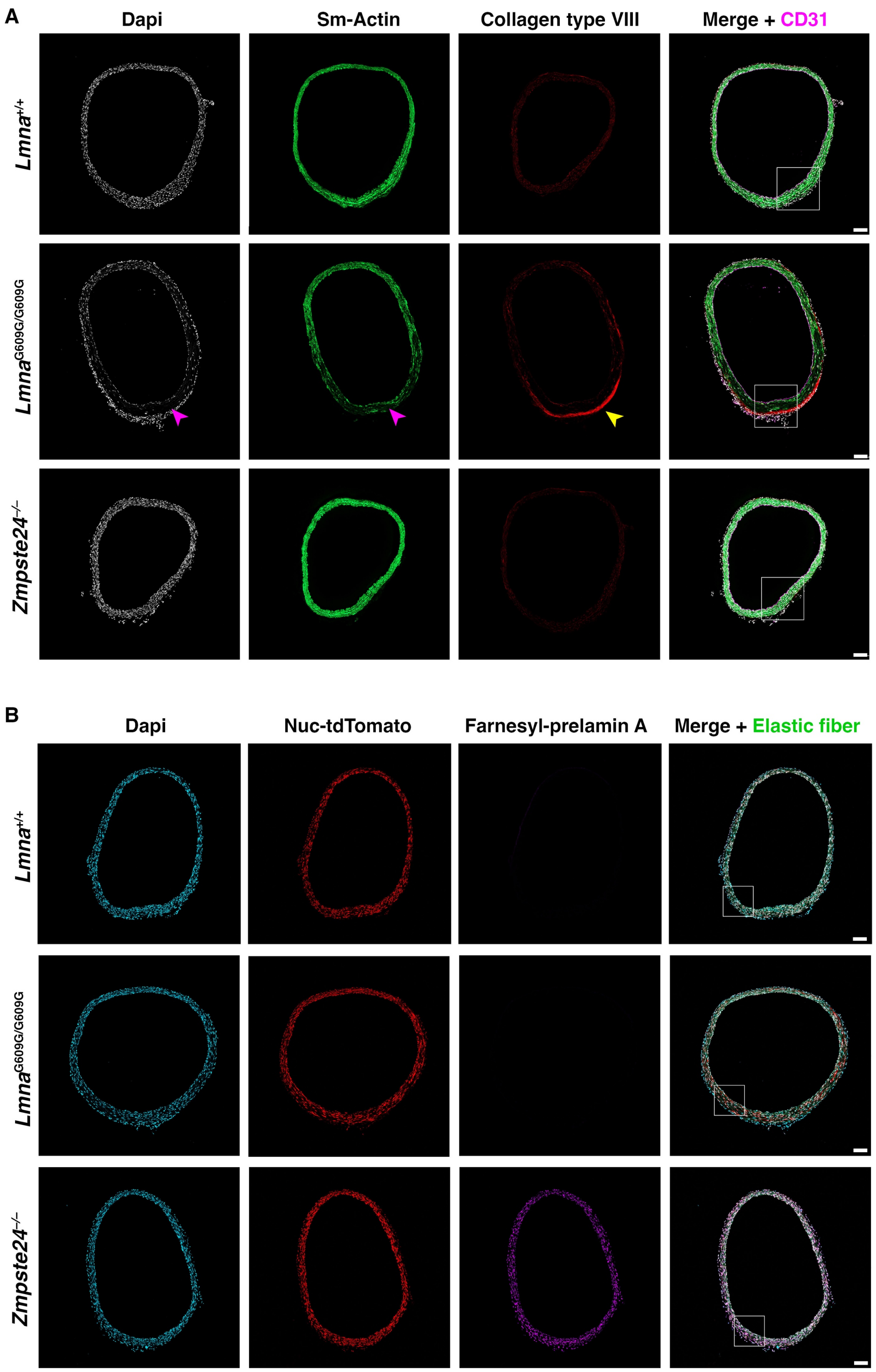

Fig. S1. SMC loss and nuclear membrane ruptures are absent in the aorta of *Zmpste24*$^{-/-}$ mice. A. Microscopy images of the proximal ascending aorta from 16-week-old *Lmna*$^{+/+}$, *Lmna*$^{G609G/G609G}$, and *Zmpste24*$^{-/-}$ mice stained with antibodies against smooth muscle actin (Sm-

bioRxiv preprint doi: https://doi.org/10.1101/2024.10.29.620896; this version posted November 3, 2024. The copyright holder for this preprint (which was not certified by peer review) is the author/funder, who has granted bioRxiv a license to display the preprint in perpetuity. It is made available under aCC-BY 4.0 International license.

actin, *green*), collagen type VIII (*red*), and CD31 (*magenta*). Nuclei were stained with Dapi (*white*). *Red* arrowheads point to areas with reduced numbers of SMC nuclei and reduced Sm-actin staining. The *yellow* arrowhead points to collagen type VIII staining in the adventitia. Scale bar, 100 µm. The boxed regions in the merged images are shown at higher magnification in Fig. 1A. **B**. Microscopy images of the proximal ascending aorta from 13-week-old *Lmna*$^{+/+}$ and *Lmna*$^{G609G/G609G}$ mice and a 21-week-old *Zmpste24*$^{-/-}$ mouse [all expressing a nuclear-targeted tdTomato (Nuc-tdTomato) transgene] stained with an antibody against farnesyl-prelamin A. The images show Dapi (*blue*), Nuc-tdTomato (*red*), farnesyl-prelamin A (*magenta*), and elastic fibers (*green*) in the merged image. Scale bar, 100 µm. The boxed regions in the merged images are shown at higher magnification in Fig. 1B.

bioRxiv preprint doi: https://doi.org/10.1101/2024.10.29.620896; this version posted November 3, 2024. The copyright holder for this preprint (which was not certified by peer review) is the author/funder, who has granted bioRxiv a license to display the preprint in perpetuity. It is made available under aCC-BY 4.0 International license.

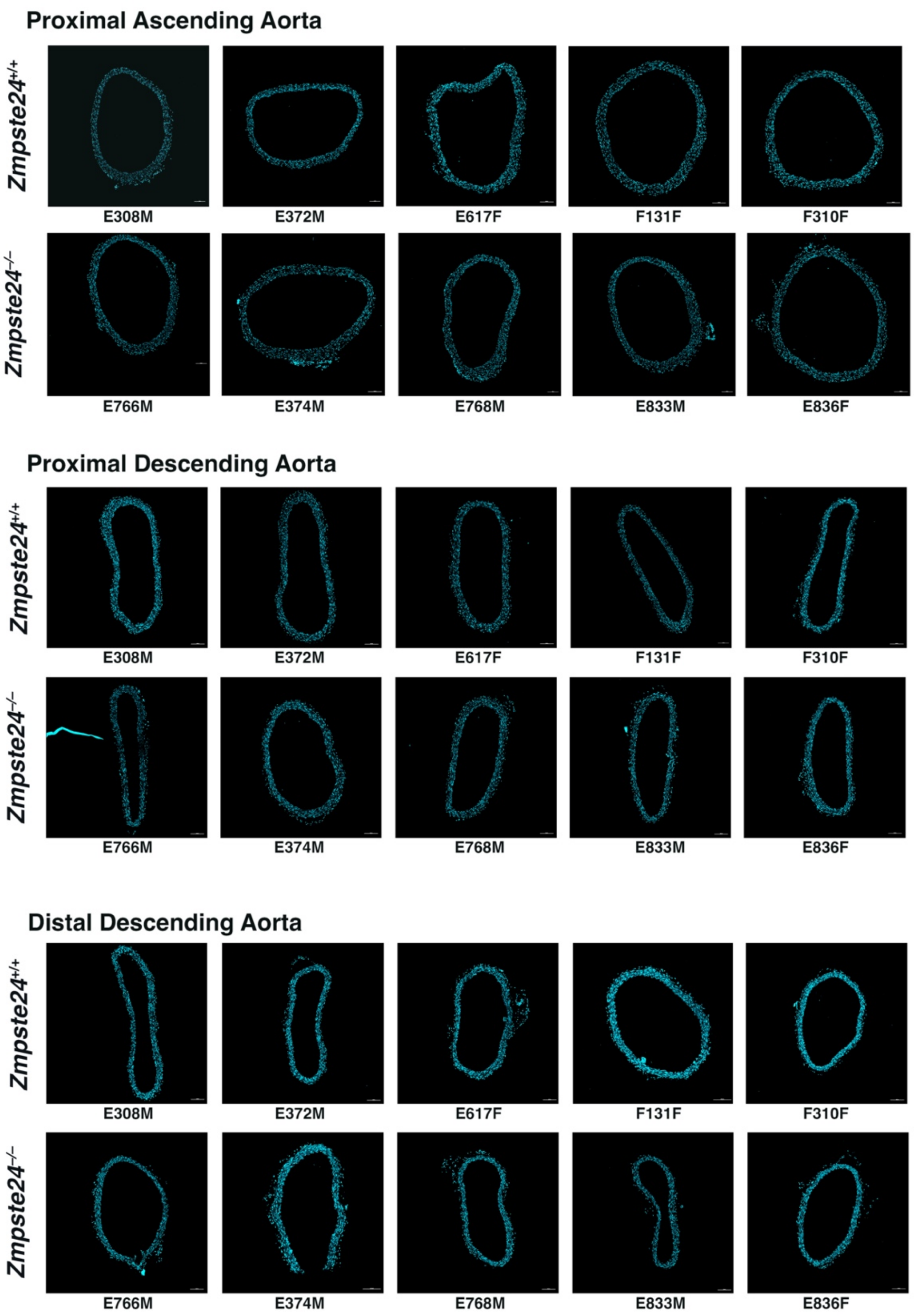

Fig. S2. Loss of smooth muscle cells is not detected in the thoracic aorta of *Zmpste24*$^{-/-}$ mice. Microscopy images of the proximal ascending, proximal descending, and distal descending aorta from 21-week-old *Zmpste24*$^{+/+}$ and *Zmpste24*$^{-/-}$ mice (5 mice/group) stained with Dapi (*blue*). The mouse IDs are shown below each image. Scale bar, 100 µm. The images of the proximal ascending aorta were used to generate the quantitative data reported in Fig. 1F.

bioRxiv preprint doi: https://doi.org/10.1101/2024.10.29.620896; this version posted November 3, 2024. The copyright holder for this preprint (which was not certified by peer review) is the author/funder, who has granted bioRxiv a license to display the preprint in perpetuity. It is made available under aCC-BY 4.0 International license.

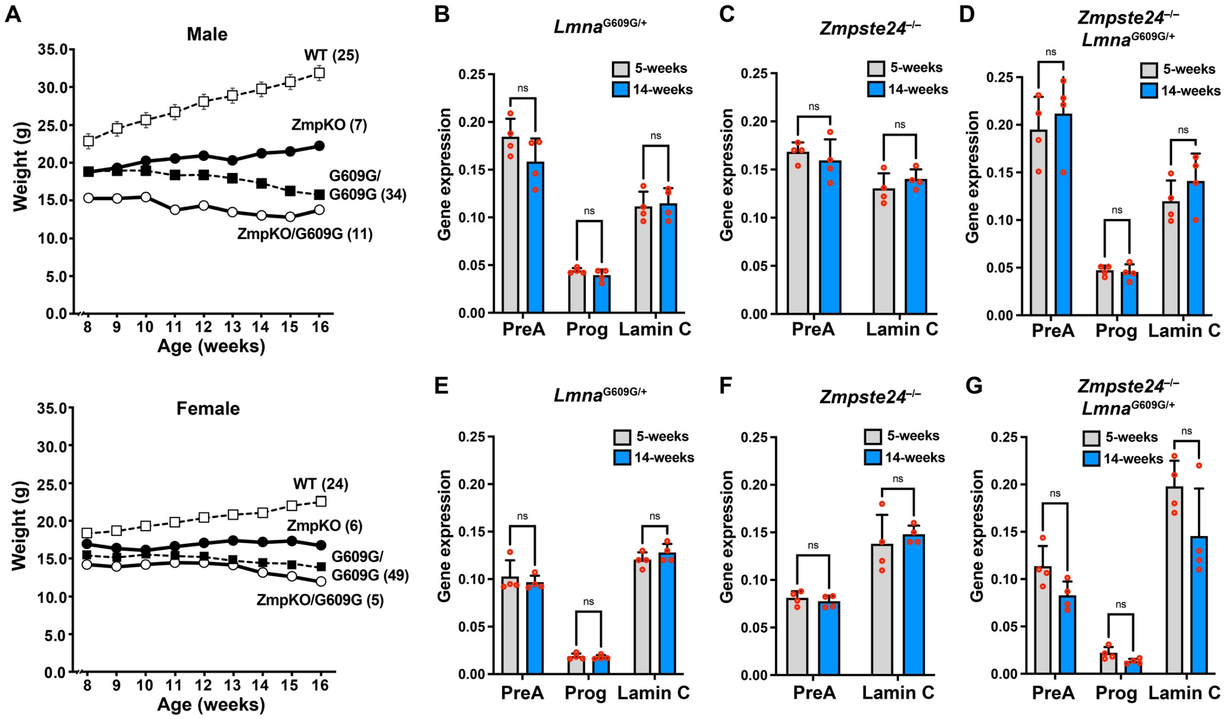

Fig. S3. Transcript levels for the A-type nuclear lamins do not increase with age in the aorta or heart of *Lmna*$^{\text{G609G/+}}$, *Zmpste24*$^{–/–}$, or *Zmpste24*$^{–/–}$*Lmna*$^{\text{G609G/+}}$ mice. A. Body weight curves for male (upper) and female (lower) *Lmna*$^{+/+}$ (WT), *Lmna*$^{\text{G609G/G609G}}$ (G609G/G609G), *Zmpste24*$^{–/–}$ (ZmpKO), and *Zmpste24*$^{–/–}$*Lmna*$^{\text{G609G/+}}$ (ZmpKO/G609G) mice. The weight curves for the *Lmna*$^{+/+}$ and *Lmna*$^{\text{G609G/G609G}}$ mice (dotted lines) are from animals generated in a different mouse colony. The numbers of mice per group are shown in parentheses. Mean ± SEM. The error bars for some data points are too small to see. **B–D.** Bar graphs showing prelamin A (PreA), progerin (Prog), and lamin C gene expression (relative to *Ppia*) in aortas from young and old *Lmna*$^{\text{G609G/+}}$, *Zmpste24*$^{–/–}$, and *Zmpste24*$^{–/–}$*Lmna*$^{\text{G609G/+}}$ mice. Mean ± SEM (n = 4 mice/group). Student's t test. ns, not significant. **E–G.** Bar graphs showing PreA, Prog, and lamin C gene expression (relative to *Ppia*) in hearts from young and old *Lmna*$^{\text{G609G/+}}$, *Zmpste24*$^{–/–}$, and *Zmpste24*$^{–/–}$*Lmna*$^{\text{G609G/+}}$ mice. Mean ± SEM (n = 4 mice/group). Student's t test. ns, not significant.

bioRxiv preprint doi: https://doi.org/10.1101/2024.10.29.620896; this version posted November 3, 2024. The copyright holder for this preprint (which was not certified by peer review) is the author/funder, who has granted bioRxiv a license to display the preprint in perpetuity. It is made available under aCC-BY 4.0 International license.

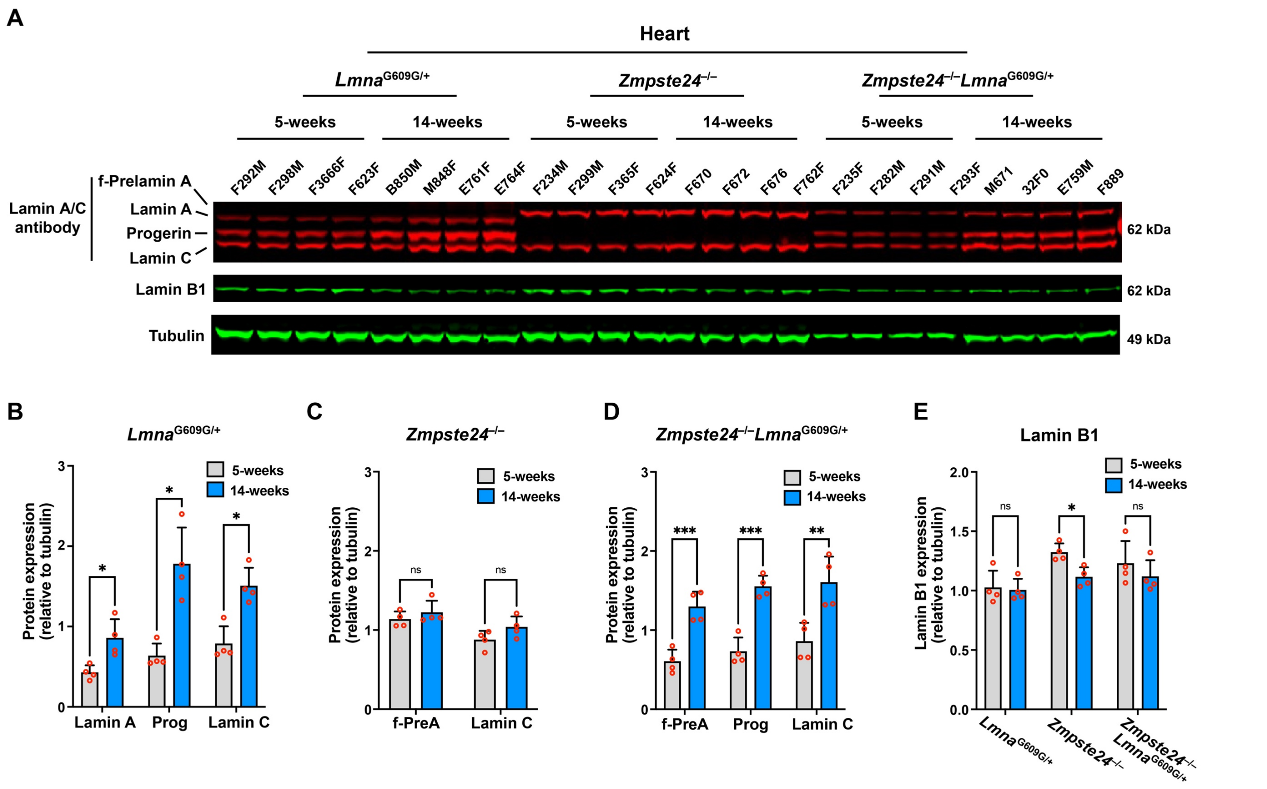

Fig. S4. Progerin causes the accumulation of the A-type nuclear lamins in the heart. A. Western blot comparing the expression of lamin A, lamin C, farnesyl-prelamin A, progerin, and lamin B1 in hearts from 5- and 14-week-old *Lmna*$^{G609G/+}$, *Zmpste24*$^{–/–}$, and *Zmpste24*$^{–/–}$*Lmna*$^{G609G/+}$ mice. Tubulin was measured as a loading control. The ages and mouse IDs are shown above each sample. **B**. Bar graph showing lamin A, progerin (Prog), and lamin C expression (relative to tubulin) in hearts from young and old *Lmna*$^{G609G/+}$ mice. Mean ± SEM (*n* = 4 mice/group). Student's *t* test. *, *P* < 0.05. **C**. Bar graph showing farnesyl-prelamin A (f-PreA) and lamin C expression (relative to tubulin) in hearts from young and old *Zmpste24*$^{–/–}$ mice. Mean ± SEM (*n* = 4 mice/group). Student's *t* test. ns, not significant. **D.** Bar graph showing f-PreA, Prog, and lamin C expression (relative to tubulin) in hearts from young and old *Zmpste24*$^{–/–}$*Lmna*$^{G609G/+}$ mice. Mean ± SEM (*n* = 4 mice/group). Student's *t* test. **, *P* < 0.01. ***, *P* < 0.001. **E.** Bar graph showing lamin B1 expression (relative to tubulin) in aortas from young and old *Lmna*$^{G609G/+}$, *Zmpste24*$^{–/–}$, and *Zmpste24*$^{–/–}$*Lmna*$^{G609G/+}$ mice. Mean ± SEM (*n* = 4 mice/group). Student's *t* test. *, *P* < 0.05. ns, not significant.

bioRxiv preprint doi: https://doi.org/10.1101/2024.10.29.620896; this version posted November 3, 2024. The copyright holder for this preprint (which was not certified by peer review) is the author/funder, who has granted bioRxiv a license to display the preprint in perpetuity. It is made available under aCC-BY 4.0 International license.

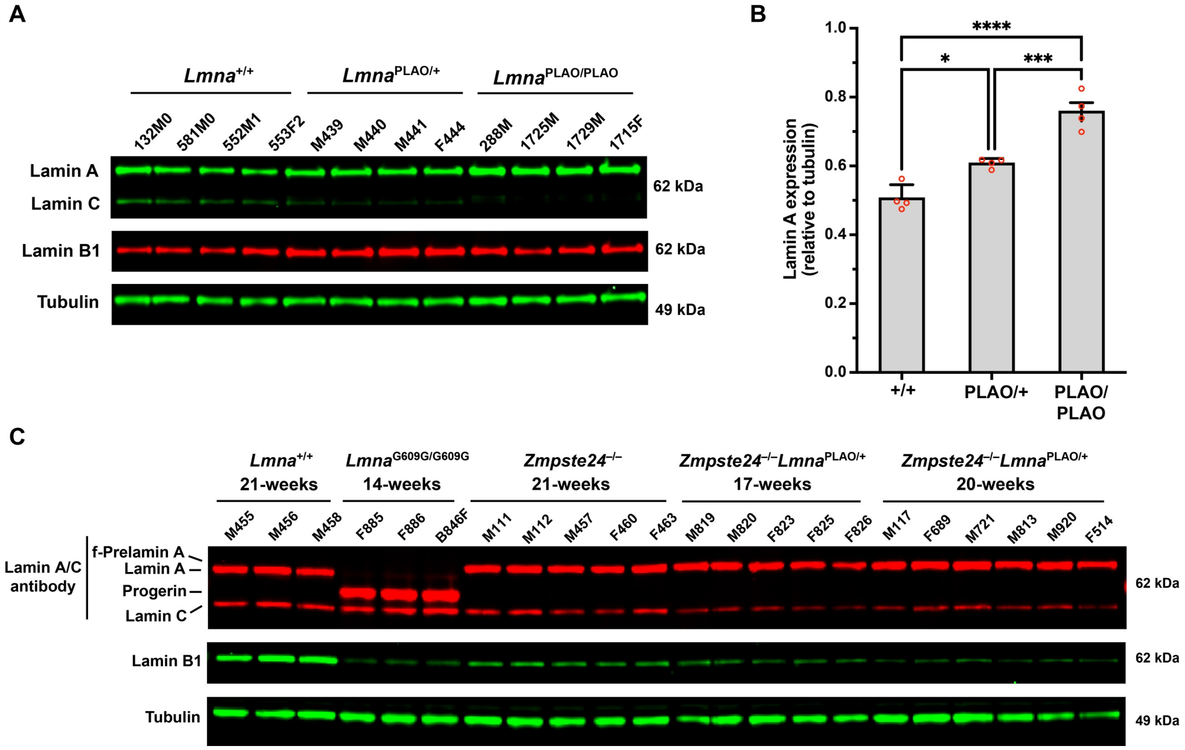

Fig. S5. The *Lmna*^{PLAO} allele increases lamin A levels in the aorta of *Zmpste24*$^{+/+}$ mice but does not increase farnesyl-prelamin A levels in *Zmpste24*$^{-/-}$ mice. A. Western blot comparing the expression of lamin A, lamin C, and lamin B1 in aortas from *Lmna*$^{+/+}$, *Lmna*$^{PLAO/+}$, and *Lmna*$^{PLAO/PLAO}$ mice. Tubulin was measured as a loading control. The mouse IDs are shown above each sample. **B**. Bar graph showing lamin A expression (relative to tubulin) in aortas from *Lmna*$^{+/+}$ (+/+), *Lmna*$^{PLAO/+}$ (PLAO/+), and *Lmna*$^{PLAO/PLAO}$ (PLAO/PLAO) mice. Mean ± SEM (n = 4 mice/group). ANOVA. *, $P < 0.05$. ***, $P < 0.001$. ****, $P < 0.0001$. **C**. Western blot comparing the expression of lamin A, lamin C, progerin, farnesyl-prelamin A, and lamin B1 in aortas from *Lmna*$^{+/+}$, *Lmna*$^{G609G/G609G}$, *Zmpste24*$^{-/-}$, and *Zmpste24*$^{-/-}$*Lmna*$^{PLAO/+}$ mice. Tubulin was measured as a loading control. The mouse IDs and ages are shown above each sample. The levels of farnesyl-prelamin A in 21-week-old *Zmpste24*$^{-/-}$ mice (n = 5) and 20-week-old *Zmpste24*$^{-/-}$*Lmna*$^{PLAO/+}$ mice (n = 6) were judged not to be different (ANOVA; P = 0.12).

bioRxiv preprint doi: https://doi.org/10.1101/2024.10.29.620896; this version posted November 3, 2024. The copyright holder for this preprint (which was not certified by peer review) is the author/funder, who has granted bioRxiv a license to display the preprint in perpetuity. It is made available under aCC-BY 4.0 International license.

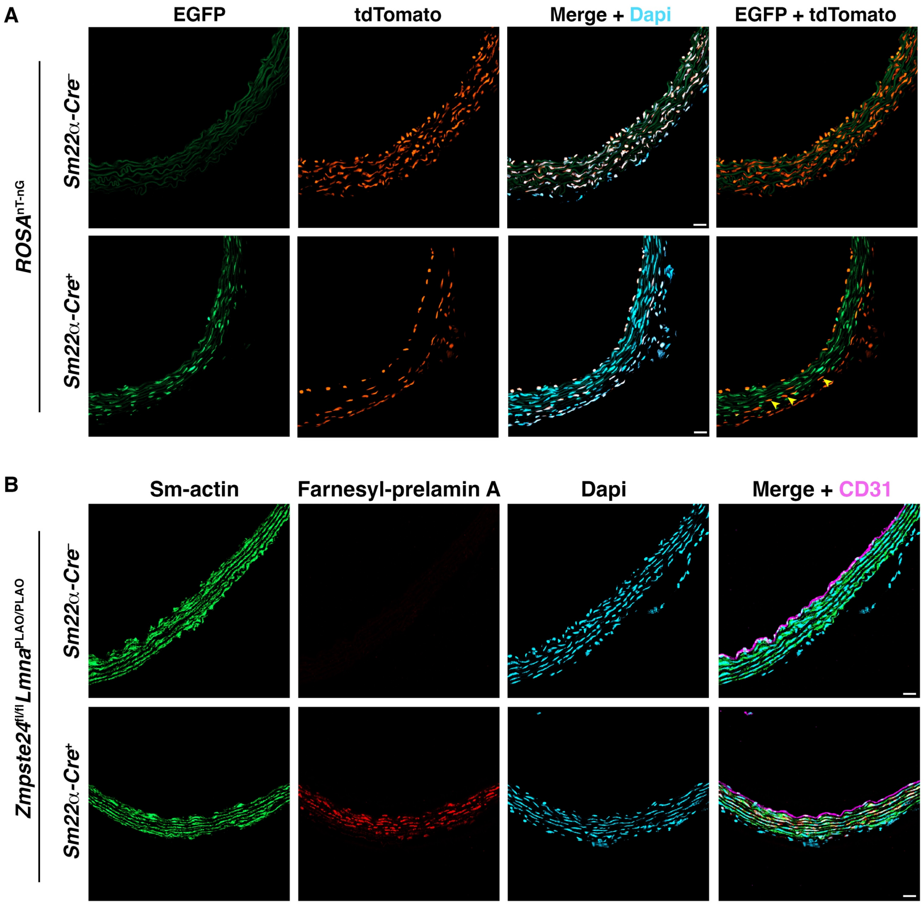

Fig. S6. Inactivation of *Zmpste24* expression in SMCs results in farnesyl-prelamin A synthesis in aortic SMCs. A. Confocal fluorescence microscopy images of the proximal ascending aorta of an 8-week-old *Sm22α-Cre*⁻*Rosa*$^{\text{nT-nG}}$ (upper row) and *Sm22α-Cre*⁺*Rosa*$^{\text{nT-nG}}$ (lower row) mouse. The *Rosa*$^{\text{nT-nG}}$ allele is a two-color reporter that expresses tdTomato in the nucleus (*39*). After *Cre* recombination, the *Rosa*$^{\text{nT-nG}}$ allele expresses EGFP, identifying cells that express *Cre*. The images show EGFP (*green*), tdTomato (*red*), and Dapi (*blue*). The *yellow* arrowheads point to SMCs in an *Sm22α-Cre*⁺*Rosa*$^{\text{nT-nG}}$ mouse that still express tdTomato, showing that *Cre* is not expressed in all aortic SMCs of *Sm22α-Cre*⁺ mice. Scale bar, 20 µm. **B.** Confocal fluorescence microscopy images of cryosections from the proximal ascending aorta of a 4-week-old *Sm22α-Cre*⁻*Zmpste24*$^{\text{fl/fl}}$*Lmna*$^{\text{PLAO/PLAO}}$ mouse (upper row) and *Sm22α-Cre*⁺*Zmpste24*$^{\text{fl/fl}}$*Lmna*$^{\text{PLAO/PLAO}}$ mouse (lower row) stained with antibodies against smooth muscle actin (Sm-actin, *green*), farnesyl-prelamin A (*red*), and CD31 (*magenta*). Nuclei were stained with Dapi (*blue*). Scale bar, 20 µm.

bioRxiv preprint doi: https://doi.org/10.1101/2024.10.29.620896; this version posted November 3, 2024. The copyright holder for this preprint (which was not certified by peer review) is the author/funder, who has granted bioRxiv a license to display the preprint in perpetuity. It is made available under aCC-BY 4.0 International license.

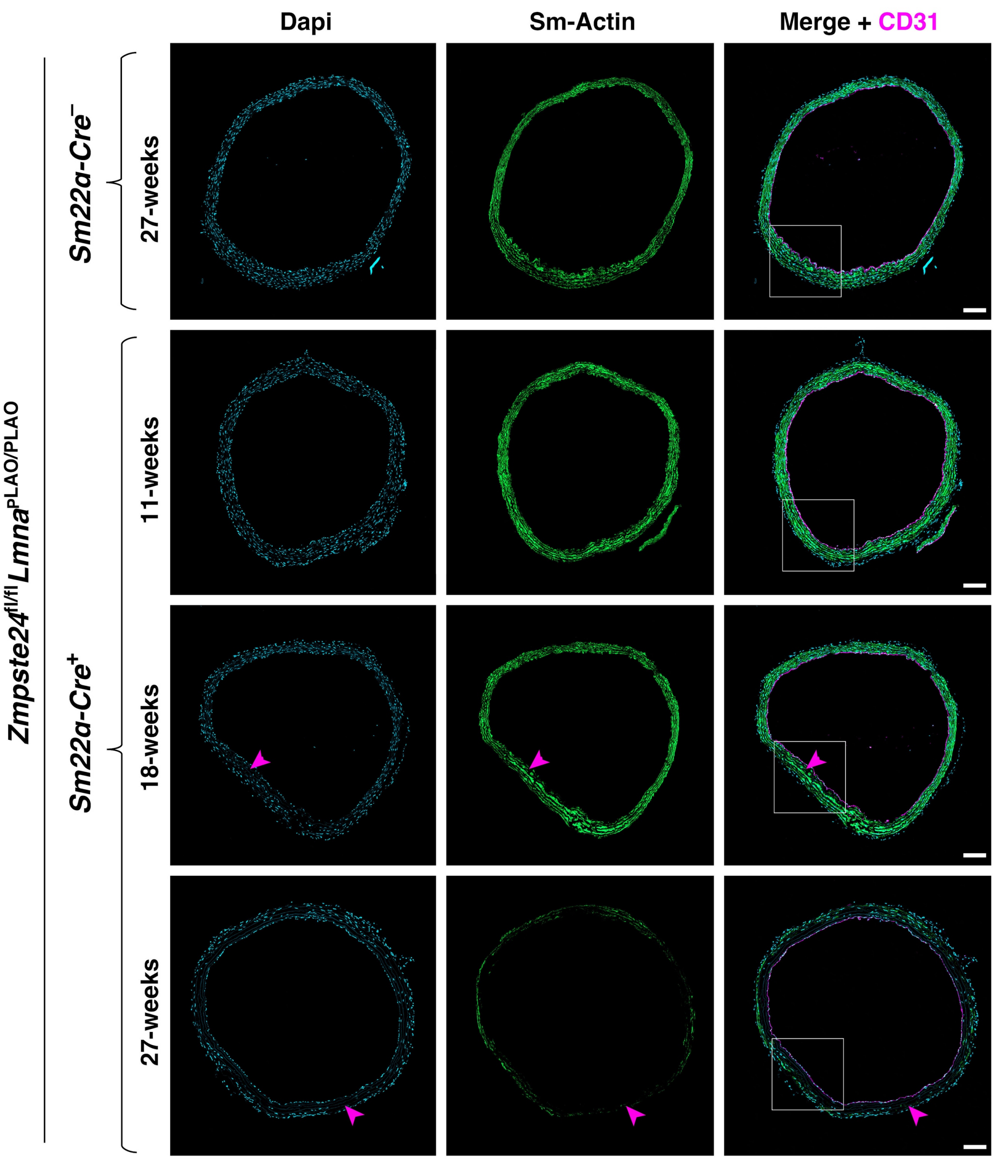

146 **Fig. S7. SMC loss and reduced smooth muscle actin staining in *Sm22α-*
147 ***Cre*$^{+}$*Zmpste24*$^{fl/fl}$*Lmna*$^{PLAO/PLAO}$ mice.** Confocal fluorescence microscopy images of the proximal
148 ascending aorta from 11-, 18-, and 27-week-old *Sm22α-Cre*$^{+}$*Zmpste24*$^{fl/fl}$*Lmna*$^{PLAO/PLAO}$ mice
149 stained with antibodies against smooth muscle actin (Sm-actin, *green*) and CD31 (*magenta*).
150 Nuclei were stained with Dapi (*blue*). As a control, images from a 27-week-old *Sm22α-Cre*$^{-}$
151 *Zmpste24*$^{fl/fl}$*Lmna*$^{PLAO/PLAO}$ mouse are shown. Scale bar, 100 μm. *Red* arrowheads point to areas
152 with reduced Sm-actin staining and reduced numbers of SMC nuclei. The boxed regions are shown
153 at higher magnification in Fig. 5C.

bioRxiv preprint doi: https://doi.org/10.1101/2024.10.29.620896; this version posted November 3, 2024. The copyright holder for this preprint (which was not certified by peer review) is the author/funder, who has granted bioRxiv a license to display the preprint in perpetuity. It is made available under aCC-BY 4.0 International license.

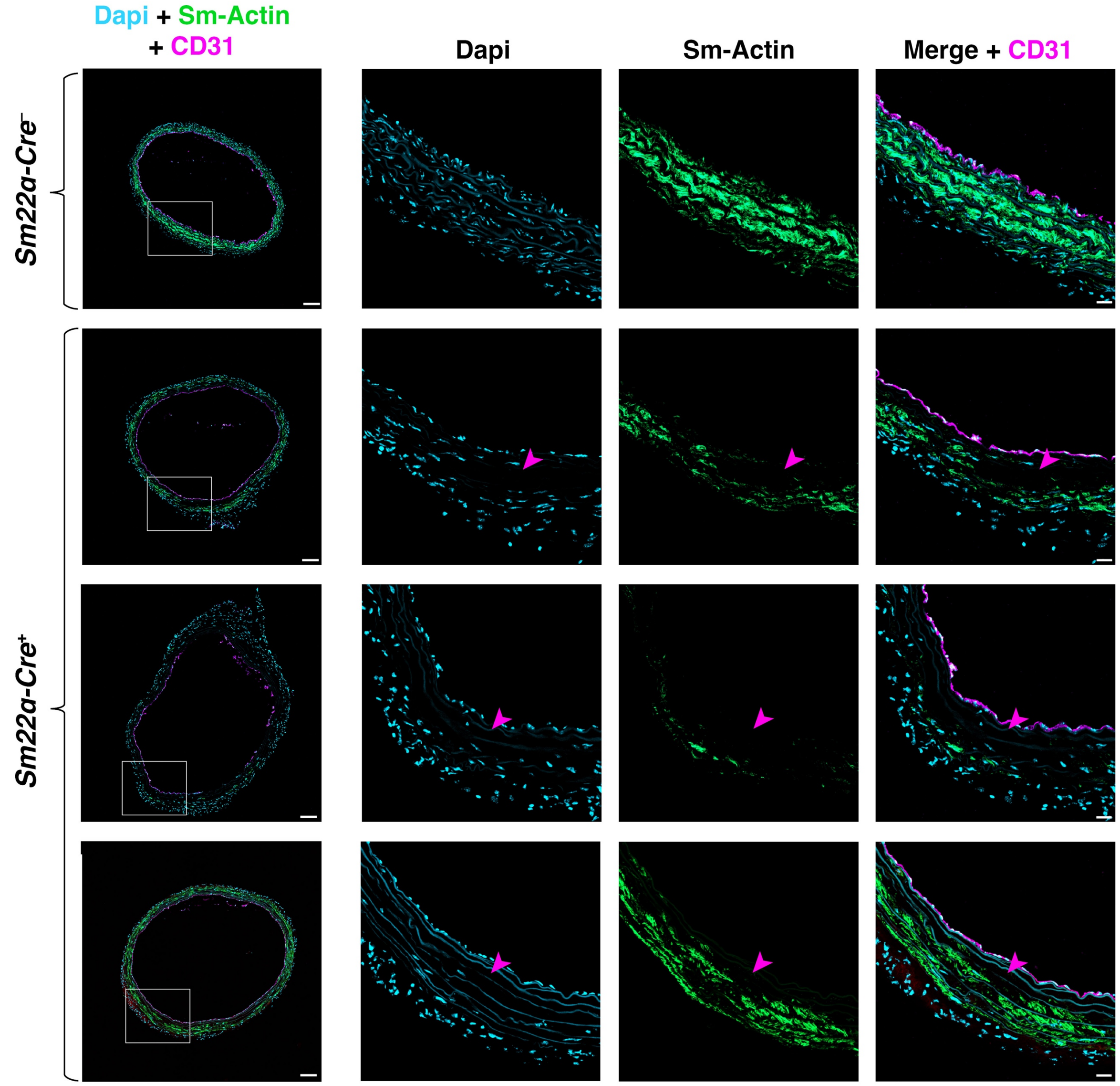

Fig. S8. Farnesyl-prelamin A causes SMC loss in the aorta of 27-week-old *Sm22α-Cre*$^{+}$*Zmpste24*$^{\mathrm{fl/fl}}$*Lmna*$^{\mathrm{PLAO/PLAO}}$ mice. Confocal fluorescence microscopy images of the proximal ascending aorta from 27-week-old *Sm22α-Cre*$^{+}$*Zmpste24*$^{\mathrm{fl/fl}}$*Lmna*$^{\mathrm{PLAO/PLAO}}$ mice stained with antibodies against smooth muscle actin (Sm-actin, *green*) and CD31 (*magenta*). Nuclei were stained with Dapi (*blue*). As a control, images from a 27-week-old *Sm22α-Cre*$^{-}$ *Zmpste24*$^{\mathrm{fl/fl}}$*Lmna*$^{\mathrm{PLAO/PLAO}}$ mouse are shown. Scale bar, 100 µm. Each row represents a different mouse. The boxed regions are shown at higher magnification to the right. *Red* arrowheads point to areas with reduced Sm-actin staining and reduced numbers of SMC nuclei. Scale bar, 20 µm.

bioRxiv preprint doi: https://doi.org/10.1101/2024.10.29.620896; this version posted November 3, 2024. The copyright holder for this preprint (which was not certified by peer review) is the author/funder, who has granted bioRxiv a license to display the preprint in perpetuity. It is made available under aCC-BY 4.0 International license.

162 **Table S1. Antibodies used for western blotting and immunocytochemistry.**
163

Antibody Description	Species	Source	Catalog #	Use	Dilution
Akt (pan)	Rabbit	Cell Signaling	4691	WB	1:1000
β-actin	Mouse	Santa Cruz Biotech	SC47778	WB	1:3000
CD31 (clone 2H8)	Hamster	DSHB	AB_2161039	IF	10 µg/ml
Collagen Type VIII	Rabbit	Antibodies-online	ABIN1718654	IF	1:20
Human lamin A	Mouse	Millipore	MAB3211	WB, IF	1:1500
Human lamin A/C	Rabbit	Abcam	Ab108595	WB, IF	1:1000
Human lamin A/C-Alexa 488 conjugated	Rabbit	Abcam	Ab185014	IF	1:1000
Lamin A/C	Mouse	Santa Cruz Biotech	SC376248	WB, IF	1:1000
Lamin B1	Goat	Santa Cruz Biotech	SC6217	WB, IF	1:1500
Lamin B1	Rabbit	Invitrogen	702972	WB, IF	1:1000
Prelamin A	Rat	In-house	clone 3C8	WB	1:1000
Phospho-Lamin A/C (Ser404)	Rabbit	Millipore Sigma	ABT1387	WB	1:500
Phospho-Akt (Ser473)	Rabbit	Cell Signaling	4060	WB	1:1000
Sm-actin	Goat	Sigma	SAB2500963	IF	1:100
Tubulin	Rat	Novus Bio	NB600-506	WB	1:3000
Anti-rabbit IR800	Donkey	LI-COR	926-32213	WB	1:10000
Anti-goat IR800	Donkey	LI-COR	926-32214	WB	1:10000
Anti-rat IR800	Donkey	ThermoFisher	SA5-10032	WB	1:5000
Anti-mouse IR800	Donkey	ThermoFisher	SA5-10172	WB	1:5000
Anti-rabbit IR680	Donkey	LI-COR	926-32221	WB	1:5000
Anti-rat IR680	Goat	LI-COR	925-68076	WB	1:5000
Anti-goat IR680	Donkey	LI-COR	926-68074	WB	1:5000
Anti-mouse IR680	Donkey	ThermoFisher	SA5-10170	WB	1:5000
Anti-mouse Alexa 488	Donkey	Invitrogen	A21202	IF	1:2000
Anti-rabbit Alexa 488	Donkey	Invitrogen	A21206	IF	1:2000

bioRxiv preprint doi: https://doi.org/10.1101/2024.10.29.620896; this version posted November 3, 2024. The copyright holder for this preprint (which was not certified by peer review) is the author/funder, who has granted bioRxiv a license to display the preprint in perpetuity. It is made available under aCC-BY 4.0 International license.

Anti-goat Alexa 488	Donkey	Invitrogen	A11055	IF	1:2000
Anti-goat Alexa 555	Donkey	Invitrogen	A21432	IF	1:2000
Anti-rabbit Alexa 555	Donkey	Invitrogen	A31572	IF	1:200
Anti-rabbit Alexa 568	Donkey	Invitrogen	A10042	IF	1:2000
Anti-mouse Alexa 568	Donkey	Invitrogen	A10037	IF	1:2000
Anti-mouse Alexa 647	Donkey	Invitrogen	A31571	IF	1:2000

bioRxiv preprint doi: https://doi.org/10.1101/2024.10.29.620896; this version posted November 3, 2024. The copyright holder for this preprint (which was not certified by peer review) is the author/funder, who has granted bioRxiv a license to display the preprint in perpetuity. It is made available under aCC-BY 4.0 International license.

Table S2. Quantitative RT-PCR primers.

Gene or transcript	Forward (5′–3′)	Reverse (5′–3′)
Ppia	TGAGCACTGGAGAGAAAGGA	CCATTATGGCGTGTAAAGTCA
Lmna	CCTATCGAAAGCTGCTGGAG	CCTGAGACTGGGATGAGTGG
Lmnb1	CAACTGACCTCATCTGGAAGAAC	TGAAGACTGTGCTTCTCTGAGC
Lamin C	GACAATGAGGATGACGACGAG	TTAATGAAAAGACTTTGGCATGG
Progerin	CTGAGTACAACCTGCGCTCA	CATGATGCTGCAGTTCTGGGAGCTCTGGAC
Prelamin A	GGTTGAGGACAATGAGGATGA	TGAGCGCAGGTTGTACTCAG
Zmpste24	CCTCTGTTTGACAAATTCACACC	AACGCTTAGATCCTTCAACAACA

bioRxiv preprint doi: https://doi.org/10.1101/2024.10.29.620896; this version posted November 3, 2024. The copyright holder for this preprint (which was not certified by peer review) is the author/funder, who has granted bioRxiv a license to display the preprint in perpetuity. It is made available under aCC-BY 4.0 International license.

Table S3. Description of cell lines.

Cell line	Modification	Source	Validation method	Mycoplasma contamination
hu-prelamin A-SMC	human (hu)-prelamin A-pTRIPZ	In-house	Western blotting and microscopy	No
hu-progerin-SMC	hu-progerin-pTRIPZ	In-house	Western blotting and microscopy	No
hu-prelamin A-SMC + nls-GFP	hu-prelamin A-pTRIPZ + nls-GFP-pCLNR	In-house	Microscopy	No
hu-progerin-SMC + nls-GFP	hu-progerin-pTRIPZ + nls-GFP-pCLNR	In-house	Microscopy	No
Zmpste24$^{-/-}$-SMC	CRISPR/Cas9 deletion of *Zmpste24*	In-house	Western blotting, qPCR and sequencing	No
Lmna$^{-/-}$-SMC	CRISPR/Cas9 deletion of *Lmna*	In-house	Western blotting, qPCR and sequencing	No
Zmpste24$^{-/-}$-SMC + hu-prelamin A-pTRIPZ	human (hu)-prelamin A-pTRIPZ	In-house	Western blotting and microscopy	No
Zmpste24$^{-/-}$-SMC + nls-GFP	*Zmpste24*$^{-/-}$ + nls-GFP-pCLNR	In-house	Microscopy	No
Zmpste24$^{-/-}$-SMC + hu-progerin	*Zmpste24*$^{-/-}$ + hu-progerin-pCLNR	In-house	Western blotting and microscopy	No
